# Development and Validation of an Extraoral Condyle Locating Device and Its Effect on Low-Level Laser Therapy in Patients With Skeletal Class II Malocclusion: Protocol for a 2-Phase Randomized Controlled Trial

**DOI:** 10.2196/94481

**Published:** 2026-10-02

**Authors:** Shilpa Kendre, Pallavi Daigavane

**Affiliations:** 1 Department of Orthodontics Datta Meghe Institute of Higher Education and Research Wardha, Maharashtra India

**Keywords:** extraoral condyle locating device, EOCLD, low-level laser therapy, LLLT, skeletal class II, myofunctional appliance therapy, condylar growth, cone beam computed tomography, CBCT, functional orthopedics, randomized controlled trial, condylar localization, laser-assisted orthodontics

## Abstract

**Background:**

Precise localization of the mandibular condyle is essential for extraoral therapeutic procedures such as low-level laser therapy (LLLT) in patients with skeletal class II malocclusion undergoing myofunctional appliance therapy. Conventional localization methods rely on palpation and visual estimation, which are operator dependent and may compromise reproducibility and treatment outcomes. To address this limitation, a spectacle-mounted extraoral condyle locating device (EOCLD) has been developed to facilitate accurate and consistent condylar targeting.

**Objective:**

This study aims to develop and validate the EOCLD for precise condylar localization and evaluate its impact on the clinical efficacy of LLLT in patients with skeletal class II malocclusion undergoing myofunctional appliance therapy.

**Methods:**

This 2-phase study will be conducted in patients aged 10 to 13 years in a growth phase with skeletal class II malocclusion. In phase 1, the EOCLD will be evaluated for accuracy and reproducibility using standardized lateral cephalometric imaging. In phase 2, a prospective randomized controlled trial will be conducted in which participants undergoing myofunctional appliance therapy will be randomly allocated to either EOCLD-guided LLLT or conventional unguided LLLT. Skeletal and dentoalveolar changes will be assessed using standardized cephalometric analyses, while condylar growth and mandibular changes will be evaluated using pretreatment and posttreatment cone beam computed tomography (CBCT) imaging. Treatment duration, patient-reported comfort, and compliance will also be assessed.

**Results:**

The study is self-funded and received approval from the institutional ethics committee on November 28, 2025. Phase 1 data collection took place from May 21 to August 30, 2026, with EOCLD validation performed in 50 participants. As of September 2026, the data are undergoing statistical analysis. Phase 2 is scheduled to commence on December 1, 2026.

**Conclusions:**

This study will establish the clinical validity of the EOCLD and determine whether guided LLLT enhances treatment efficiency and skeletal outcomes in skeletal class II therapy.

**Trial Registration:**

Clinical Trials Registry–India CTRI/2026/02/102982; https://tinyurl.com/4t3bwrnz

**International Registered Report Identifier (IRRID):**

PRR1-10.2196/94481

## Introduction

Despite the fact that most oral health issues are avoidable, millions of people worldwide suffer from them, including malocclusion. Malocclusion is a morphological variation, not a disease, that may or may not be linked to pathological diseases. It is a common oral pathology that is typically listed third among global public health priorities for dental diseases, after dental caries and periodontal disease [1-3].

An individual’s facial appearance has a lasting effect on their well-being. Unacceptable dental appearance has frequently been linked to detrimental effects on one’s ability to succeed in at work, accept oneself in peer groups, and maintain a positive self-image. Children with extremely severe or crippling malocclusion may be diagnosed, and corrective measures should be implemented as soon as possible to avoid a widespread negative influence on their psychological development [2,4].

Early disease detection and prevention can reduce the economic burden on the nation and decrease the need for more expensive treatment options. Orthodontic treatment at an early age frequently includes modifying a child’s facial growth to create a more harmonious interaction between various facial components. Growth accelerations and decelerations occur periodically during normal human development. Orthodontists are particularly interested in these times of rapid growth since the adolescent growth spurt, when various facial bones are growing at a favorable rate, is the period during which growth changes are best performed. When therapy is started when the patient’s skeleton is maturing at its ideal stage, positive results can be anticipated with the fewest side effects [5-8].

Boys and girls experience the pubertal growth peak at different times; thus, it would be ideal to conduct separate analyses for boys and girls. However, prior research has provided combined findings for male and female samples and has been unable to offer a thorough examination of the duration of the peak of pubertal development across the 3 skeletal classes [8-12].

There has been much discussion about the appropriate age and time to begin orthodontic therapy. Specifically, there is a valid question as to whether a dual-phase treatment administered during occlusal development is preferable to a single-phase treatment administered at a later age when the mouth is fully developed, particularly when the malocclusion is associated with skeletal discrepancies resulting from altered jaw growth**.** However, orthodontic treatment is frequently administered at a young age [13-15].

When undergoing orthodontic treatment, the way teeth move is determined by how they react to the forces used in the orthodontic process, which remodel the periodontal tissues, particularly the alveolar bone. Accelerating alveolar bone remodeling during treatment is crucial to reducing the usual 2- to 3-year treatment period, which can be taxing for patients [16].

Tissues can be affected by laser radiation in a number of ways, from photodisruption to biostimulation. The tissue’s rising effect is contingent upon both the energy density and the duration of irradiation. “Biostimulating effects” refer to the effects of laser radiation that do not cause a local temperature increase in tissues of more than 1 °C. “Low-level laser therapy” (LLLT) refers to treatments that use the biostimulation potency of laser radiation to achieve their goals [16,17].

Since 1971, scientists have been investigating how low-level laser use affects biostimulatory processes. Laser radiation can stimulate a wide range of processes, including wound healing, bone regeneration, nerve regeneration, collagen synthesis, fibroblast and chondrocyte proliferation, and wound healing. Biostimulation is a useful technique in dentistry that helps heal aphthous ulcers, repair bone in certain periodontal defects, and speed up the process of osteointegration following implantation [18-20].

Low-level laser radiation has applications in orthodontics that include reducing postadjustment pain [8], promoting tooth movement, and promoting bone regeneration in the midpalatal suture area following rapid maxillary expansion [21,22].

For precise and effective application of LLLT in the temporomandibular joint (TMJ) region, accurate localization of the mandibular condyle is crucial. Traditionally, anatomical landmarks and palpation techniques have been used to estimate the condylar position, but these methods lack precision. Any deviation in laser targeting may reduce its therapeutic efficacy or cause unintended tissue effects.

An extraoral condyle locating device (EOCLD) may significantly enhance the accuracy of LLLT delivery by providing real-time guidance on condylar positioning. The EOCLD can help clinicians pinpoint the condyle’s position noninvasively, ensuring optimal laser penetration and potentially enhancing therapeutic effects on condylar growth An EOCLD may significantly enhance the accuracy of LLLT delivery by providing real-time guidance on condylar positioning. The EOCLD can help clinicians pinpoint the condyle’s position noninvasively, facilitating more precise laser targeting of the condylar region and potentially enhancing the therapeutic effects on condylar growth, mandibular length, and glenoid fossa adaptation. This technological advancement may improve treatment outcomes and minimize variability in laser application, especially across different growth patterns and gender groups. Although the use of an EOCLD for LLLT applications is not extensively documented, existing research underscores the essential role of an EOCLD in enhancing the effectiveness of LLLT in promoting condylar growth and mandibular advancement.

## Methods

### Study Design

The study will be conducted in 2 phases. In Phase 1 (clinical validation of EOCLD), an observational, cross-sectional validation study will assess the accuracy and reproducibility of the newly developed Extraoral Condyle Locating Device (EOCLD) for noninvasive mandibular condyle localization. In Phase 2: (randomized controlled trial), a randomized controlled trial will compare the clinical efficacy of EOCLD-guided versus conventional Laskin-guided low-level laser therapy (LLLT) in skeletal Class II patients undergoing myofunctional appliance therapy.

### Study Setting

This study will be conducted at the Department of Orthodontics and Dentofacial Orthopedics, Sharad Pawar Dental College and Hospital, Wardha, Maharashtra, India, which is an academic dental teaching institution. The study is designed as a single-center prospective investigation and will be carried out in 2 phases within the same institutional setting.

#### Phase 1

The EOCLD has been developed as a chairside guiding aid to facilitate standardized and reproducible localization of the mandibular condyle during extraoral LLLT. The device has been designed using stable extraoral anatomical landmarks to improve the precision of condylar localization and reduce operator-dependent variability during laser application.

Phase 1 of the study, involving the development and validation of the EOCLD (Figure 1), will be conducted in the clinical and radiographic facilities of the Department of Orthodontics and Dentofacial Orthopedics. Initially, EOCLD-guided condylar localization will be performed using conventional Laskin reference points in the first 10 participants. On the basis of radiographic assessment of localization accuracy, a modified extraoral condylar reference location will be identified and standardized, if required, to optimize localization precision.

**Figure 1:**
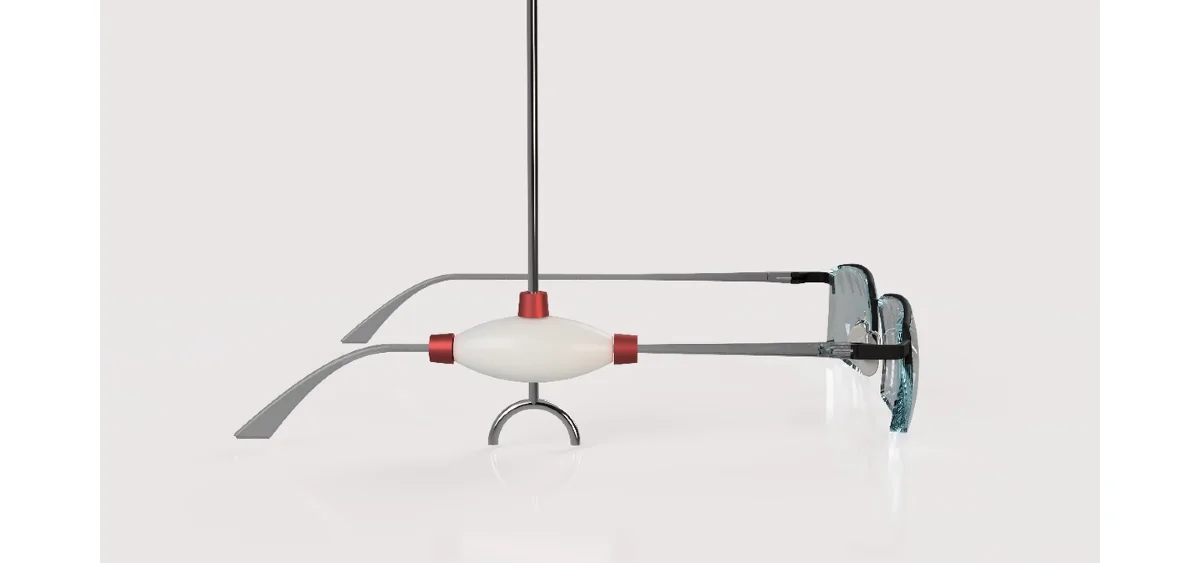
Extraoral condyle locating device (EOCLD).

Following refinement of the localization protocol, the finalized EOCLD-guided method will be evaluated in the remaining participants. Validation will be performed by comparing EOCLD-guided localization points with the condylar position identified on standardized lateral cephalometric radiographs. Localization accuracy will be assessed by measuring the linear deviation between the clinically localized point and the radiographic condylar position.

Reproducibility of EOCLD-guided localization will be assessed using repeated measurements to evaluate intraoperator and interoperator reliability. Additional parameters, including the time required for condylar localization and the need for repeated localization attempts, if any, will also be recorded to assess procedural feasibility and consistency.

#### Phase 2

Phase 2, consisting of the randomized controlled trial, will be conducted in the outpatient orthodontic clinic of the same department. Eligible growing patients with skeletal class II malocclusion undergoing myofunctional appliance therapy will be screened, recruited, and followed up according to the study protocol. All clinical examinations, appliance fabrication and delivery, LLLT procedures, radiographic assessments, and follow-up evaluations will be performed under standardized institutional conditions by trained personnel. Pretreatment and posttreatment cone beam computed tomography (CBCT) imaging and lateral cephalometric radiographs will be obtained in phase 2 for assessment of condylar growth and mandibular skeletal changes. The single-center study setting is intended to ensure uniformity in diagnosis, intervention delivery, and outcome assessment throughout the study period.

### Study Population

#### Phase 1

Participants aged 10 to 35 years who are visiting the Department of Orthodontics for treatment will be included in phase 1.

#### Phase 2

Growing patients aged 10 to 13 years with skeletal class II malocclusion who are visiting the Department of Orthodontics for treatment will be included in phase 2.

### Participant Enrollment

Potential participants reporting to the outpatient clinic of the Department of Orthodontics and Dentofacial Orthopedics for correction of skeletal class II malocclusion will be screened consecutively during the study period. Initial screening will include detailed clinical examination, medical and dental history taking, growth assessment, and radiographic evaluation using lateral cephalometric analysis to confirm skeletal class II diagnosis and eligibility for myofunctional appliance therapy.

Participants fulfilling the predefined inclusion and exclusion criteria (Textbox 1) will be considered eligible for enrollment. The study procedure, objectives, benefits, and possible risks will be explained in detail to the participants and their parents or legal guardians. Written informed consent from parents or legal guardians, along with participant assent wherever applicable, will be obtained prior to enrollment.

For phase 1, eligible participants will undergo EOCLD-guided condylar localization and radiographic assessment for validation of the device. In phase 2, eligible participants will be recruited into the randomized controlled trial and allocated to either the EOCLD-guided LLLT group or the conventional unguided LLLT group according to the randomization protocol. Participant recruitment, follow-up scheduling, appliance delivery, laser therapy sessions, and outcome assessments will be performed under standardized institutional conditions throughout the study period.

**Inclusion and exclusion criteria.**

**Inclusion criteria**

***Phase 1***

Participants will be eligible for phase 1 if they meet all of the following criteria:

- Aged 10 to 35 years
- No history of orthodontic or orthopedic treatment
- Normal temporomandibular joint (TMJ) function and no pain or clicking

***Phase 2***

Participants will be eligible for phase 2 if they meet all of the following criteria:

- Aged 10 to 13 years and in the active growth phase
- Diagnosed with skeletal class II malocclusion due to mandibular retrusion
- An A point–nasion–B point (ANB) angle of 4° to 8° and a Sella–nasion–B point (SNB) angle of ≤78°
- An overjet of ≥5 mm
- In the prepubertal or pubertal growth stage, confirmed using a hand-wrist radiograph or cervical vertebral maturation index stage 2 to 4
- No history of orthodontic or orthopedic treatment
- Willingness to undergo cone beam computed tomography (CBCT) and lateral cephalogram imaging
- Cooperative behavior, with informed consent obtained from a parent or legal guardian and assent obtained from the participant

**Exclusion criteria**

***Phase 1***

Participants will be excluded from phase 1 if they meet any of the following criteria:

- Craniofacial anomalies or TMJ pathology
- Systemic conditions affecting bone growth
- A history of facial trauma or surgery in the TMJ region

***Phase 2***

Participants will be excluded from phase 2 if they meet any of the following criteria:

- Craniofacial syndromes, cleft lip or palate, or congenital facial deformities
- A history of orthodontic or orthopedic treatment
- Systemic diseases (eg, growth hormone disorders or juvenile arthritis) affecting growth
- TMJ disorders, pain, or restricted mouth opening at baseline
- Poor oral hygiene, active dental caries, or periodontal disease
- Uncooperative behavior or inability to follow the study protocol
- Contraindications to low-level laser therapy, such as photosensitivity or epilepsy, or use of medications affecting bone metabolism
- Inability or refusal to undergo CBCT imaging
- Metal allergy or hypersensitivity to appliance materials

### Sample Size Considerations

Phase 1 will include 50 samples. For phase 2, the effect size obtained from phase 1 will be used to calculate the minimum number of participants required to achieve 80% statistical power at a 5% significance level (α=.05). An additional 10% to 15% will be added to compensate for possible dropouts or attrition.

### Data Collection

Pretreatment and posttreatment TMJ CBCT scans and lateral cephalograms will be obtained for all participants to evaluate condylar growth and mandibular skeletal changes. All radiographic records will be securely stored in a password-protected cloud-based system to ensure data integrity, confidentiality, and accessibility for analysis. A comprehensive database will be maintained in both digital (Microsoft Excel based) and hard-copy formats, documenting participant details and EOCLD-guided condylar localization records in the EOCLD-assisted LLLT group. In the EOCLD-assisted LLLT group, condylar localization will be performed using the EOCLD, with cephalometric confirmation prior to laser application to enhance localization accuracy.

### Randomization, Allocation Concealment, Blinding, and Sample Size (Phase 2)

#### Intervention and Control Procedures

All participants in phase 2 will receive standardized twin block myofunctional appliance therapy fabricated and delivered according to a uniform clinical protocol. Participants will be instructed to wear the appliance full time throughout the treatment period.

LLLT will be administered bilaterally over the condylar region using a 940 nm diode laser device operating at 0.3 W. In the intervention group, laser application will be guided using the EOCLD-confirmed condylar localization point, while in the control group, laser application will be performed using conventional Laskin reference points. Laser irradiation will be delivered with an energy dose of 5 J per point for 20 seconds according to the predefined application schedule. Participants will receive 3 sessions per week during the first month, followed by 1 session per week during the second and third months, totaling 16 sessions.

#### Treatment Adherence Monitoring

Compliance with twin block appliance wear and scheduled laser therapy sessions will be monitored at each follow-up visit through clinical assessment, patient reporting, and appointment records. Required appliance adjustments and activations will be performed according to the standardized treatment protocol.

#### Safety and Adverse Event Reporting

Any adverse events, discomfort, soft tissue irritation, TMJ symptoms, appliance breakage, or complications related to laser therapy, radiographic imaging, or myofunctional appliance therapy will be documented throughout the study period. Appropriate clinical management will be provided whenever necessary.

#### Data Management

Participant information and radiographic records will be coded to maintain confidentiality. Clinical records, cephalometric measurements, CBCT images, and EOCLD localization data will be securely stored in password-protected digital databases and institutional records accessible only to the research team.

#### Handling of Withdrawals and Missing Data

Participants will be free to withdraw from the study at any stage without affecting their ongoing treatment. Reasons for withdrawal, loss to follow-up, or protocol deviations will be documented. Missing data will be handled using appropriate statistical methods, including intention-to-treat analysis and multiple imputation wherever applicable.

### Trial Timeline

Phase 1 of the study will involve EOCLD development, refinement, and validation procedures. Following completion of phase 1 and finalization of the sample size calculation, phase 2 recruitment and randomized allocation will be initiated. Participants will undergo active myofunctional appliance therapy and LLLT for 9 months, followed by posttreatment radiographic and clinical evaluation. Data analysis and dissemination of findings will be performed after completion of participant follow-up.

### Intervention Protocol

#### LLLT Protocol

LLLT will be used as an adjunct to myofunctional appliance therapy in phase 2 of the study. Laser irradiation will be delivered bilaterally to the TMJ or condylar region using a 940 nm diode laser with an output power of 0.3 W.

In the intervention group, the site of laser application will be determined using the EOCLD to achieve anatomically guided localization of the mandibular condyle. In the control group, laser application will be performed using the conventional Laskin method for condylar localization. Each application point will receive 5 J of energy over 20 seconds, delivered bilaterally under standardized operating conditions. All procedures will be performed by the same trained operator to minimize interoperator variability and maintain consistency in laser administration.

The laser treatment schedule will consist of 3 sessions per week during the first month, followed by 1 session per week during the second and third months, resulting in a total of 16 sessions over the intervention period. All laser procedures will be performed by the same trained operator under standardized conditions to minimize procedural variability.

#### Myofunctional Appliance Protocol

All participants will receive a twin block appliance as part of standard functional orthopedic therapy for the correction of skeletal class II malocclusion. The appliance will be fabricated using a standardized construction protocol and delivered by the same operator to ensure consistency across participants. Participants will be instructed to wear the appliance full time, except during eating and oral hygiene procedures. Follow-up visits will be scheduled in accordance with the laser application schedule to monitor appliance fit, treatment compliance, and clinical progress. Any required adjustments or activations will be performed according to a standardized appliance management protocol. The total duration of twin block therapy will be 9 months, and all participants will receive the same appliance protocol irrespective of group allocation, ensuring that the only variable between groups is the method of laser localization.

### Outcome Measures and Statistical Analysis

#### Outcome Measures

This 2-phase study includes both validation outcomes (phase 1) and clinical outcomes (phase 2).

#### Phase 1 Outcomes

The primary outcome in phase 1 will be the accuracy of condylar localization, assessed by measuring the linear deviation (in millimeters) between the EOCLD-guided localization point and the condylar position identified on standardized lateral cephalometric radiographs. This assessment will be performed at the time of EOCLD validation and radiographic comparison.

Secondary outcomes in phase 1 will include intraoperator and interoperator reproducibility assessed using repeated localization measurements, the time required for condylar localization, and the need for repeated localization attempts, if any. In the initial 10 participants, the EOCLD will be evaluated using conventional Laskin reference points to identify and standardize a modified extraoral condylar reference location, if required. The comparative accuracy, reproducibility, and clinical feasibility of EOCLD localization using conventional and modified reference locations will subsequently be assessed.

#### Phase 2 Outcomes

The primary outcome in phase 2 will be the change in effective mandibular length following treatment, assessed using standardized lateral cephalometric analysis at completion of active treatment (T2). A clinically meaningful difference between the EOCLD-guided LLLT group and the conventional unguided LLLT group will be considered during sample size calculation and statistical analysis.

Secondary outcomes in phase 2 will include changes in additional skeletal parameters such as the Sella–nasion–point B (SNB) angle and condylar growth-related measurements; dentoalveolar changes, including overjet reduction and molar relationship correction; treatment duration required to achieve predefined clinical correction; and patient-centered outcomes, including comfort and treatment acceptability. Posttreatment stability, where applicable, will also be evaluated.

For phase 1, all validation outcomes will be assessed during EOCLD testing and radiographic comparison procedures. For phase 2, outcome assessments will be performed at baseline before initiation of treatment (T0), during active treatment at 3 months (T1), and at completion of active treatment (T2).

### Statistical Analysis

Statistical analysis will be performed using appropriate statistical software. Descriptive statistics will be calculated for all study variables and expressed as means and SDs, medians and IQRs, and ranges, as appropriate. Normality of continuous variables will be assessed using the Shapiro-Wilk test.

For phase 1, agreement between EOCLD-guided and Laskin reference point–based condylar localization will be evaluated using paired *t* tests or Wilcoxon signed-rank tests, depending on the data distribution. Agreement and measurement error will additionally be assessed using Bland-Altman analysis with predefined acceptable error limits. Reliability and reproducibility will be evaluated using intraclass correlation coefficients (ICCs) for intraoperator and interoperator measurements.

For phase 2, all analyses will be performed on an intention-to-treat basis, with all randomized participants included in the final analysis. Baseline comparability between groups will be assessed using the independent *t* test, Mann-Whitney *U* test, chi-square test, or Fisher exact test, as appropriate. The primary end point will be skeletal mandibular changes assessed at the completion of functional appliance therapy. Repeated measurements obtained at baseline, during treatment, and at posttreatment follow-up will be analyzed using repeated measures ANOVA (RM-ANOVA) or linear mixed-effects models, depending on the data structure and completeness of follow-up. These models will evaluate group effect, time effect, and group×time interaction while adjusting for relevant covariates, including age, sex, and baseline skeletal measurements.

Missing data and participant dropouts will be documented. Where appropriate, missing data will be managed using multiple imputation or mixed model estimation under the assumption that the data are missing at random. Per-protocol sensitivity analyses may also be performed to assess the robustness of the findings. Statistical significance will be set at *P*<.05.

### Radiation Safety

In phase 1, standardized lateral cephalometric radiographs will be used to assess the clinical and radiographic reproducibility of the EOCLD and to evaluate its approximation to the condylar region relative to conventional orthodontic radiographic landmarks. This phase is intended as a preliminary validation under routine clinical conditions rather than as 3D anatomical confirmation.

In phase 2, lateral cephalometric radiographs will be obtained as part of routine orthodontic diagnostic and treatment records. In addition, pretreatment and posttreatment CBCT scans will be performed to evaluate condylar positional and morphological changes associated with myofunctional appliance therapy and LLLT, wherever clinically justified and ethically approved.

Considering the inclusion of a growing pediatric population aged 10 to 13 years, radiation exposure will be carefully justified and minimized throughout the study. All imaging procedures will be performed only when clinically and scientifically necessary and not solely for research convenience. Radiographic investigations will follow the ALARA (as low as reasonably achievable) principle and, where applicable, the ALADAIP (as low as diagnostically acceptable being indication-oriented and patient-specific) concept. The smallest feasible field of view, minimum diagnostically acceptable exposure settings, and standardized pediatric imaging protocols will be used during CBCT acquisition. Appropriate protective measures will also be used wherever applicable to minimize unnecessary radiation exposure.

### Ethical Considerations

This study will be conducted in accordance with the principles of the Declaration of Helsinki, applicable institutional ethical guidelines, and relevant national regulations governing research involving human participants. Ethics approval for this study has been obtained from the institutional ethics committee of Sharad Pawar Dental College, Datta Meghe Institute of Higher Education and Research, Sawangi (Meghe), Wardha (DMIHER(DU)/IEC/2025/598).

As the study involves a pediatric population, written informed consent will be obtained from the parents or legal guardians of all participants prior to enrollment, along with age-appropriate assent from the children. Participation will be voluntary, and participants will have the right to withdraw from the study at any stage without affecting their ongoing treatment. Data confidentiality and participant privacy will be maintained throughout the study.

### Dissemination Plan

The findings of this study will be disseminated through publication in peer-reviewed journals and presentations at national and international scientific conferences.

## Results

The study is self-funded and received approval from the institutional ethics committee on November 28, 2025. Results will be reported following completion of data collection and statistical analysis. Phase 1 data collection took place from May 21 to August 30, 2026, with EOCLD validation performed in 50 participants. As of September 2026, the data are undergoing statistical analysis. Phase 2 is scheduled to commence on December 1, 2026. Data collection and radiographic assessment for EOCLD validation have been completed, and the resulting data are currently undergoing statistical analysis. The analysis will evaluate the accuracy and reproducibility of extraoral condylar localization using the EOCLD in comparison with the predefined radiographic reference. Phase 2, comprising the randomized controlled clinical evaluation of EOCLD-guided versus non–EOCLD-guided low-level laser therapy in skeletal class II patients undergoing myofunctional appliance therapy, is planned following completion of the phase 1 analysis and is expected to commence in 3 to 4 months. Results from phase 1 are expected to be submitted for publication in 2027.

## Discussion

### Anticipated Findings

This study is designed to evaluate the clinical utility of the EOCLD as a novel, noninvasive tool for improving the precision and reproducibility of condylar localization during LLLT in patients with skeletal class II malocclusion undergoing myofunctional appliance therapy. It is anticipated that EOCLD-guided laser application may improve localization accuracy, reduce operator-dependent variability, and contribute to the more standardized delivery of adjunctive laser therapy.

Accurate localization of the mandibular condyle remains a significant challenge in extraoral therapeutic procedures. Conventional landmark-based approaches depend largely on palpation and operator experience, which may lead to variability in laser application and inconsistent therapeutic outcomes. The present 2-phase study has therefore been designed to address this limitation through device validation in phase 1 and randomized clinical evaluation in phase 2.

Previous studies investigating LLLT in orthodontics and functional orthopedic therapy have reported variable findings, partly due to differences in laser application protocols and difficulties in accurately identifying the condylar region. The EOCLD has been conceptualized as a reproducible extraoral guiding aid intended to standardize condylar localization and improve consistency of laser delivery. The findings of the present study may therefore help clarify the potential role of precise condylar targeting in enhancing treatment outcomes.

A major strength of this protocol is its 2-phase design, combining methodological validation with clinical evaluation through a randomized controlled trial. Additional strengths include standardized myofunctional appliance therapy, predefined outcome measures, and structured statistical analysis. However, the study may have certain limitations, including its single-center setting, moderate sample size, and the challenges associated with radiographic assessment in a pediatric population.

Future multicenter studies with larger sample sizes and longer follow-up periods may be required to confirm the long-term stability and generalizability of the findings. Following study completion, the results will be disseminated through peer-reviewed scientific publications and presentations at national and international scientific meetings.

Overall, this study is expected to provide preliminary evidence regarding the feasibility and clinical applicability of EOCLD-guided LLLT and may contribute to improving the precision and standardization of laser-assisted functional therapy in orthodontic practice.

### Conclusions

Accurate and reproducible localization of the mandibular condyle is essential for improving the consistency of extraoral therapeutic procedures such as LLLT. The proposed EOCLD may provide a standardized, noninvasive approach for condylar localization and has the potential to reduce operator-dependent variability during laser application. If found to be clinically reliable, this approach may contribute to improved precision and standardization of laser-assisted functional therapy in patients with skeletal class II malocclusion. The findings of this study may also provide a foundation for further research on image-guided and device-assisted adjunctive therapies in orthodontics.

## Abbreviations

ALADAIP: as low as diagnostically acceptable being indication-oriented and patient-specific
ALARA: as low as reasonably achievable
ANB: A point–nasion–B point
CBCT: cone beam computed tomography
EOCLD: extraoral condyle locating device
ICC: intraclass correlation coefficient
LLLT: low-level laser therapy
RM-ANOVA: repeated measures ANOVA
SNB: Sella–nasion–B point
TMJ: temporomandibular joint

## Data Availability

No data are associated with this protocol.
